# Detection of IgG antibody during the follow-up in patients with COVID-19 infection

**DOI:** 10.1186/s13054-020-03138-4

**Published:** 2020-07-20

**Authors:** Jiao Liu, Jun Guo, Qianghong Xu, Guolong Cai, Dechang Chen, Yanfei Shen

**Affiliations:** 1 Department of Critical Care Medicine, Ruijin Hospital, Shanghai Jiao Tong University School of Medicine, No.197 Ruijin 2nd Road, Shanghai, China; 2 Department of Critical Care Medicine, Ruijin Hospital North, Shanghai Jiao Tong University School of Medicine, No.999 Xiwang Road, Shanghai, China; 3 Department of Critical Care Medicine, Huazhong University of Science and Technology Union Jiangbei Hospital, 111 Success Avenue, Wuhan, China; 4 Department of Critical Care Medicine, Zhejiang Hospital, No. 12, Linyin Road, Hangzhou, 310000 Zhejiang People’s Republic of China

Although most patients with COVID-19 in China have been cured and discharged, we noticed a small proportion of these patients had re-positive RT-PCR test during the follow-up period [1]. The causes of this re-infection remain unclear. In common COVID-19 cases [2], both the IgM and IgG antibodies significantly increased within a short period. However, in a case series report [3], the IgG was relatively low in re-infected COVID-19 cases. Thus, we investigated the IgG status in recovered patients during the follow-up period.

This retrospective study was performed in Wuhan JiangBei Hospital, China. COVID-19 infection was confirmed by the RT-PCR test. The IgM and IgG antibodies were detected using colloidal immunization methods. Only cured patients were included in this analysis. Patient consent was waived due to the retrospective nature. The ethics committee of Wuhan JiangBei Hospital approved this study.

During follow-up, only simple tests were performed, such as blood routine examination, antibody test, and chest computed tomography (CT). For accuracy, missing data were not imputed.

Continuous variables were presented as mean ± standard deviation, and Student’s *t* test was used unless indicated. Categorical data were compared using the chi-square test. *p* < 0.05 was considered statistically significant. All statistical analyses were performed using STATA 14.0.

We studied 484 patients with positive IgG, the minimum period from onset to IgG detection was 10 days, and the maximum period was 100 days (Fig. 1). Meanwhile, 18% of these patients had negative IgG results, and this was confirmed by more than two IgG tests in 37 patients. The mean duration from onset to IgG test was close between positive and negative IgG groups (50.5 ± 14.8 vs. 43.3 ± 15.0, days).

**Fig. 1:**
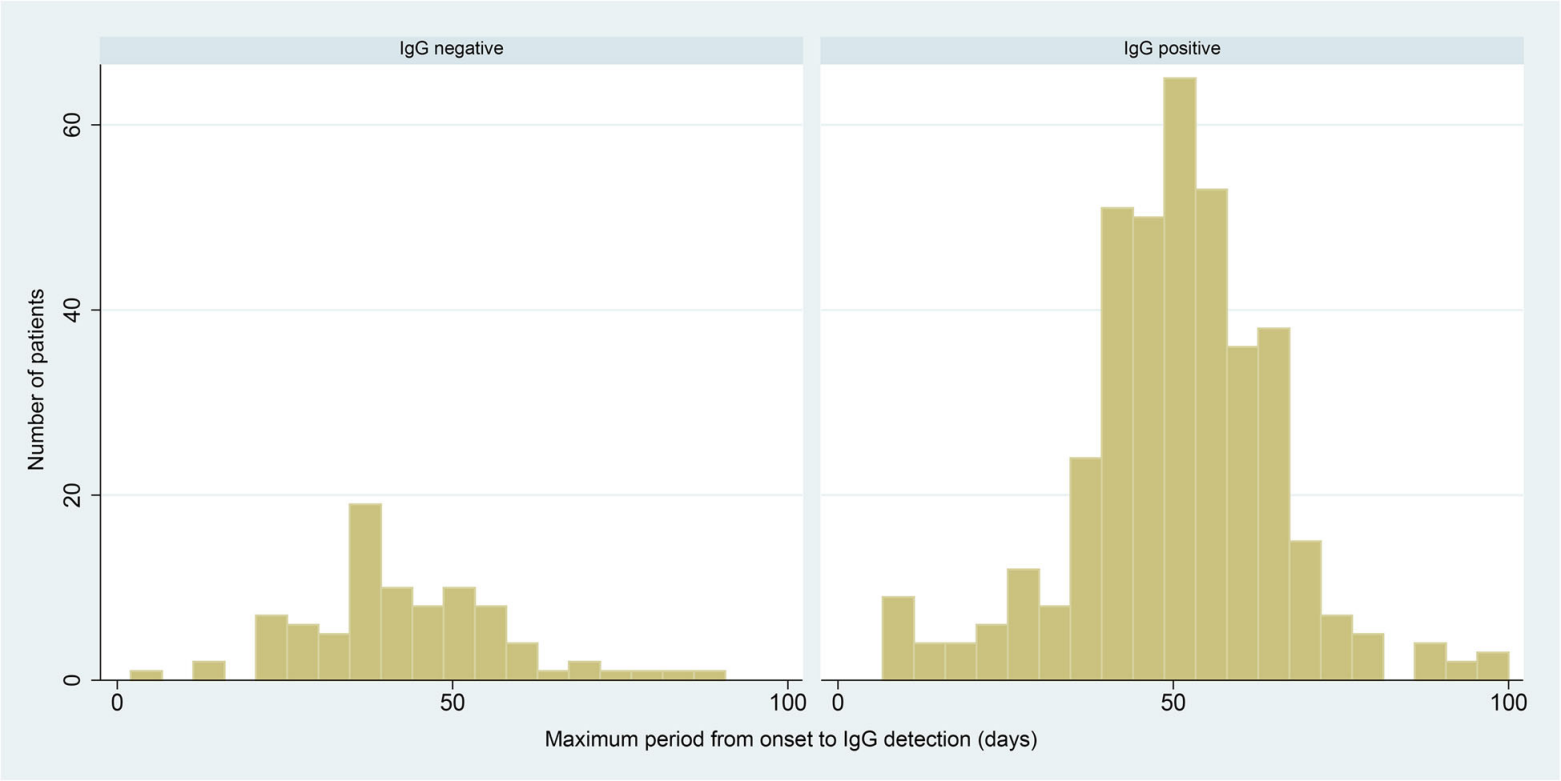
Maximum period from disease onset to IgG detection in the negative and positive IgG groups

Further, compared to the negative IgG group, both the lymphocyte (1.3 ± 0.0 vs. 1.6 ± 0.1, *p* = 0.001) and neutrophil counts (3.5 ± 1.6 vs. 5.0 ± 3.0, *p* <  0.001) were lower in the positive group. Besides, the percent of abnormal CT findings at follow-up was higher in the positive IgG group (259/372 vs. 22/64, *p* <  0.001) (Table 1).

**Table 1:** Comparisons between patients with positive and negative IgG antibody

Variables	Positive IgG (*n* = 397)	Negative IgG (*n* = 87)	Negative IgG (≥ 2 tests) (*n* = 37)	*p*
Age (years)	51.2 ± 13.9	49.6 ± 17.2	51.8 ± 19.4	0.365
Gender (male, %)	190 (47.8)	43 (49.4)	13 (35.1)	0.791
White blood cell count on admission	5.3 ± 1.8	7.1 ± 3.1	6.9 ± 2.7	< 0.001
Lymphocyte count on admission	1.3 ± 0.0	1.6 ± 0.1	1.6 ± 0.7	0.001
Neutrophil count on admission	3.5 ± 1.6	5.0 ± 3.0	4.7 ± 2.5	< 0.001
White blood cell count at follow-up	6.3 ± 1.7	6.5 ± 1.8	6.2 ± 1.8	0.387
Lymphocyte count at follow-up	2.1 ± 0.6	2.1 ± 0.6	2.0 ± 0.7	0.738
Neutrophil count at follow-up	3.7 ± 1.4	3.9 ± 1.4	3.6 ± 1.3	0.296
Maximum duration of IgG test	50.5 ± 14.8	43.3 ± 15.0	50.6 ± 12.1	< 0.001
Maximum duration of IgG test*, median (min and max value)	51 (10–100)	42 (2–90)	50 (28–90)	< 0.001
Abnormal CT findings at follow-up^#^ (which indicate residual infection)	259/372	22/64	10/32	< 0.001

All comparisons were made between positive IgG and negative IgG groups

*IgG* immunoglobulin G, *CT* computed tomography

*Presented as median (minimum and maximum value), compared using rank-sum test

^#^Any chest CT findings that suggested residual infection during follow-up were defined as abnormal

Re-infection with COVID-19 in recovered patients has been occasionally encountered in clinical practice. Weak evidence [3] indicated that the IgG level was low in these re-infected COVID-19 cases. As IgG plays a critical role in immune response, understanding IgG status in recovered patients is necessary for preventing re-infections. In the current study, we found that 18% of the recovered patients had negative IgG. The mechanism remains unclear. However, we also found that compared to the positive group, the lymphocyte on hospital admission was higher in the negative group. Evidence [4, 5] has indicated that lymphocyte count is an independent predictor for COVID-19 severity. Thus, we inferred that compared to patients with positive IgG, those with negative IgG might have relatively mild COVID-19 infection, and the slight impact on their immune system leads to the higher lymphocyte and negative IgG during the follow-up period. This hypothesis was also supported by the CT finding that the residual infection on chest CT disappears more quickly in patients with negative IgG. If this is the case, the risk of re-infection of COVID-19 in these patients should be carefully assessed in the later stage of epidemic prevention.

This study was limited by the qualitative IgG tests and short follow-up period. Further study should focus on the time-dependent change of the antibody level and the identification of those who are still at risk of re-infection in recovered patients.

## Data Availability

All the data were available from the corresponding author on a reasonable request.
